# Electric Transmission and Distribution Network Air Pollution

**DOI:** 10.3390/s24020587

**Published:** 2024-01-17

**Authors:** Saverio De Vito, Antonio Del Giudice, Girolamo Di Francia

**Affiliations:** ENEA—Italian National Agency for New Technologies, Energy and Sustainable Economic Development, P.le E. Fermi, 1, 80055 Napoli, Italy; saverio.devito@enea.it (S.D.V.); antonio.delgiudice@enea.it (A.D.G.)

**Keywords:** battery, electric distribution, electric transmission, pollution, power cable, sensor, switch, transformer

## Abstract

There is a consensus within the scientific community regarding the effects on the environment, health, and climate of the use of renewable energy sources, which is characterized by a rate of harmful polluting emissions that is significantly lower than that typical of fossil fuels. On the other hand, this transition towards the use of more sustainable energy sources will also be characterized by an increasingly widespread electrification rate. In this work, we want to discuss whether electricity distribution and transmission networks and their main components are characterized by emissions that are potentially harmful to the environment and human health during their operational life. We will see that the scientific literature on this issue is rather limited, at least until now. However, conditions are reported in which the network directly causes or at least promotes the emissions of polluting substances into the environment. For the most part, the emissions recorded, rather than their environmental or human health impacts, are studied as part of the implementation of techniques for the early determination of faults in the network. It is probable that with the increasing electrification of energy consumption, the problem reported here will become increasingly relevant.

## 1. Introduction

Climate and environmental emergencies are increasingly drawing worldwide attention to the need to increase the rate of renewable energy sources (RES) in the global energy mix [1]. In 2022, electricity represented about 38% of the global energy mix, but since more than 60% of it was produced by fossil fuels, its real contribution in terms of an environmental and climate friendly source was around just 15% [2]. As a matter of fact, in the same year, the overall percentage of RES in terms of global world energy consumption was only just over 14%; it is worth mentioning here that this rate is expected to become higher than 60% in 2050 [3]. The growing use of renewable energy sources brings about environmental benefits that are effectively measurable in terms of a reduction in climate-changing gas emissions and, more generally, in a decrease in polluting substances [4]. For example, from 2005 to 2018, despite a global increase in primary energy consumption of around 44%, a threefold increase in the use of renewables made it possible to obtain a net decrease of almost 130 kt in the concentrations of all major pollutants, and the major impact on this effect can be attributed to photovoltaic and wind power diffusion [4,5].

Car electrification is expected to play a key role in this scenario. Although a large abatement of NO_x_, CO, CO_2_, and VOC exposure can be obtained by the introduction of electric cars at a fast pace, fine particulate exposure cannot be reduced comparatively due to non-exhaust emissions (brake, tyre, and road wear), which represent the most relevant particulate pollution sources [6]. Furthermore, tyre-related particulate emissions could increase due to a possible increase in electric car weight. Despite this, an overall gas pollutant exposure from private transport of close to zero can be foreseen [7,8].

The replacement of fossil fuels with renewable energy sources directly generating electricity calls for the substantial implementation and strengthening of the electric transmission and distribution network, as well as for the replacement of any machine or equipment based on fossil fuels with others based on electric propulsion. One of the results of such a transformation is that power lines, cables, transformers, substation equipment, and charge storage apparatus are expected to correspondingly increase both in number and in dimensions. How deeply this process would impact human lives and the quality of the environment in which we live is not completely known. Certainly, the use of lower temperature energy processes will bring benefits both in terms of the CO_2_ footprint and a decrease in the concentrations of the pollutants specifically connected to the use of fossil fuels [9,10]. Nevertheless, the energy transition also involves aspects that, if not decidedly negative, are still questionable and whose overall environmental impact deserves to be investigated in greater detail. The first is related to the energy and environmental costs of production, recycling, and recovery of the raw materials that make it possible [11]. However, another aspect that needs to be considered is that the quantitative increase in transmission lines, substations, batteries, etc. will lead to a corresponding increase in sources that can modify the environment both in terms of physical and chemical polluting agents. With regard to the former, these effects are mainly related to electromagnetic pollution and acoustic noise. As far as the former is considered, from a strictly scientific point of view, the effect of electromagnetic fields on the health of living organisms may still be considered as an open research topic although low-frequency magnetic fields (ELF-MF), typical for example of transmission lines, have already been classified by the International Agency for Research on Cancer (IARC) as possibly carcinogenic, and recent studies have confirmed their possible negative role on health [12,13,14]. With regard to acoustic noise, intensity levels in the order of tens of decibels, perfectly detectable by those who live near electrical substations, were reported by Piana and Roozen and mainly attributed to mechanical effects [15]. Even more interesting is the case of the noise generated by high voltage lines that can reach several tens of decibels at the source, which is known to be correlated with the corona effect [16].

In this paper, we discuss a topic that has been less addressed, up to now, in the scientific and technical literature: electricity transmission and distribution networks as atmospheric chemical polluting agents. In Section 2, the network components, their malfunctions, and specific emissions are reported, and in Section 3 the commercial solutions available for the various network components are reported. Finally, a discussion of the results is presented in Section 4, and general conclusions are drawn in Section 5.

## 2. Network Components, Malfunctions, and Specific Emissions

**Figure 1:**
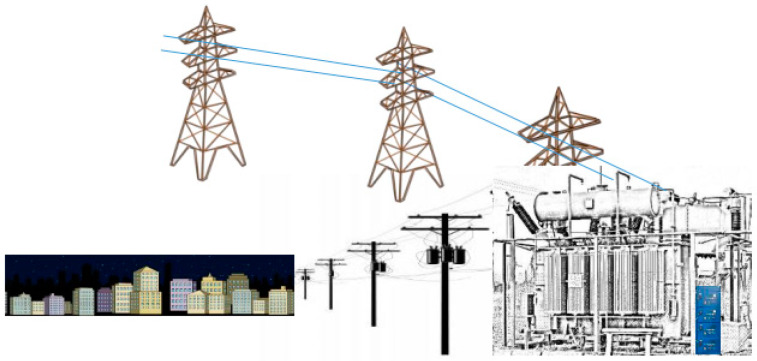
The general structure of an electric transmission and distribution network with its main components: the HVPL cables that carry the electricity from a power plant to a transformer, the switch cabinets, and the LVPLs carrying energy to the city.

In this section, the connections between emissions and normal or faulty operating conditions in different elements of the power transport, distribution, and use network are reviewed. Figure 1 schematically shows the main components of an electric transmission and distribution network: the HVPL cables carry the electricity from an electric power plant to a transformer, and through the use of proper switchgears, LVPLs eventually carry the energy underground to cities. In general, such a system is already equipped with various monitoring sensors [17]. A network of this type is normally assumed to be characterized by practically zero emissions in terms of air polluting substances during its operation, except when malfunctions are observed in one or more of the components that characterize the network itself. Consequently, chemical sensors are only referred to as some specific components. The scientific literature reporting how these components, during the operating lifetime and in case of malfunction, may emit one or more gaseous components will be reported and discussed with the aim of understanding if the emitted pollutant concentrations can be correlated with a specific failure.

In this scenario, the possibility of using arrays of solid-state sensors to monitor the operating status of the aforementioned components and prevent the onset of serious anomalies in the network is discussed. This paragraph is structured in sub-paragraphs, each of which focuses on a specific network component: Section 2.1 is related to transmission and distribution cables; Section 2.2 is related to transformation substations and distribution panels; Section 2.3 is related to switches; and Section 2.4 is related to batteries.

### 2.1. Transmission and Distribution Lines

**Figure 2:**
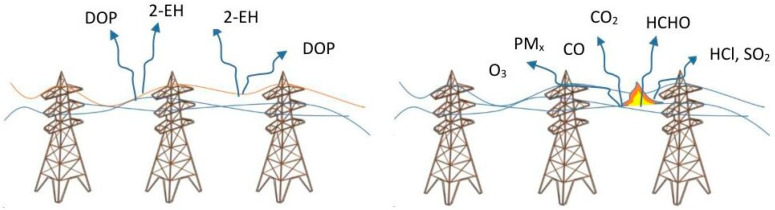
Polluting emissions in case of power lines overheating and the corona effect (**left**) and cables burning (**right**).

During the operating lifetime of an electrical transmission and distribution power line, its components show signs of physical fatigue due to ageing wear in particular operating conditions or specific unexpected damage. At present, in the EU, the electric grid extends for more than 10^6^ km and is expected to strongly increase in length because of the foreseen massive electrification. Due to such a large and pervasive diffusion, power lines and cables can be considered as the most pervasive source of air pollution in terms of toxic emissions into the atmosphere in cases of both overheating and fire. This last occurrence is, of course, the most dangerous. Burjupati and Arunjothi, for example, studied the toxic emissions associated with cable fires and concluded that even lethal concentrations of CO, CO_2_, HCHO, HCl, and SO_2_ can be reached in a burning event [18]. The main problem is obviously related to the combustion of the polymers and the various additives used for cable fabrication, with the burning of plastic components being well known in the literature as a source of toxic compounds at concentrations associated with this type of phenomenon, which are always very high. A detailed list of 40 pollutants released during such a burning event is reported by Ortner and Hensler [19]. It is worth noting here that such a problem is also correlated to any of the plastic-based components, such as the plastic cabinets used throughout the network. In case of cables overheating, dioctyl phthalate (DOP) and 2-ethylhexanol (2-EH) are normally released well before the burning event. Interestingly enough, an increased risk of overheating has also been reported for underground cables used for distribution networks if they partly come from overhead cables, especially in the case of solar exposition [20]. From the point of view of air pollution, both in case of overheating and in case of burning, the most appropriate equipment to detect specific air pollutants is FTIR, XPS, gas chromatography, and other similar techniques. These tools have the disadvantage of being expensive, complex, and unsuitable for continuous analysis, as Densley points out in Ref. [21]. However, in the case of overheating of electrical cables, solid-state monitoring-based solutions have recently been proposed that allow for the development of equipment suitable for more distributed monitoring. Han and coworkers, for example, proposed MOX sensors for the detection of DOP and 2-EH compounds generally present in the overheating phenomena of many electrical cables [22]. Although the work does not provide any evidence on the concentrations of the gases released during the overheating event and subsequently detected, the authors conclude that the solid-state sensors are suitable for detecting the presence of DOP and 2-EH well before the onset of fire phenomena. This technique can therefore be useful for preventing fire damage. Liu and coworkers compared both commercial and homemade sensor arrays for the same purpose to detect DOP, 2-EH, and benzene [23]. In addition, in this study, the concentrations of the target gases are not reported. The work concludes, similarly to Han and coworkers, that the investigated technique allows for the detection of DOP and 2EH well before the ignition of fire in electrical cables. A similar conclusion was reached by Knoblauch and co-authors [24], who studied a solid-state sensor array based on SnO_2_ with different additives. The applied system is shown to be able to detect toxic components (CO, propylene) even before the cables show changes in color due to overheating. From the point of view of the concentrations emitted, one of the few relevant studies concerns the emission of toxic gases from the ageing of electrical cables used in nuclear power plants (and therefore subject to particularly restrictive regulations). This study shows how, in the case of twenty-year ageing structures, CO, HCl, and HBr concentrations that exceed the permitted limits are measured, whereas in the case of SO_2_, a sensible increase in gas emission over time is observed. In conclusion, a significant increase in the overall toxicity index proposed for the system under examination can be measured in any case [25]. Transmission networks exhibit peculiar properties related to their HV operation. High-voltage power lines (HVPLs) may in fact behave as a source of charged aerosols and ions. Specifically, corona effects in HVDC lines usually generate atomic oxygen and other radicals, including OH or ions, mostly through the ionization of nitrogen molecules and their compounds. Deposition of particulate matter or dirt generally enhances the effect and hence ion production [26]. The production of ions and aerosols poses environmental and safety issues because it can add to inhalable pollutants in directly exposed populations, including HVPL maintenance workers [27,28]. Recently, Jung et al. implemented a measurement campaign in 2019 to establish correlations between aerosol concentrations and operative parameters near HVPLs. Their results confirmed that measured concentrations are correlated with operative conditions and specifically with current and magnetic fields, whereas a high correlation was found between fine particulate (equal or smaller than 10 um) concentrations and humidity [29]. It is worth noting that under extreme weather conditions, elastic extension or fatigue failures in conductors, poles, crossarms, or surrounding objects (trees) have been found to be responsible for wildfire ignition, generating volatiles whose detection can act as an alarm trigger [30]. Because of the corona effect, HVPLs are found to be sources of atmospheric pollutants such as ozone and nitrogen oxides [31]. Therefore, power transmission networks can be considered as a collection of connected linear sources of air pollution. It is extremely difficult to quantify the actual absolute (and hence relative) impact on the recorded pollution levels, which may vary locally in space and time due to accumulation and transport patterns depending on weather and topographic conditions [32]. Elansky et al. showed that ozone levels near 220 kV powerlines are 2 ppb higher than background recordings, whereas an average excess of 2.5 ppb to 4.6 ppb was recorded at multiple sites near 500 kV powerlines [33]. They also attempted an overall evaluation, revealing that emissions by high-voltage power transmission lines during the 1990s could have globally accounted for 400–600 × 10^3^ tons/year, at least 0.1% of ozone tropospheric formation by photochemical processes. Local exposure has also been investigated in other studies. Cociorva and co-authors quantified the potential additional intake of pollutants in the area of HVPLs through ad hoc measurement campaigns. Peak ozone and nitrogen oxide concentrations were found to exceed levels measured in the surrounding background areas by 13% and 30%, respectively [31]. Interestingly, hourly concentration patterns reported anomalous peaks during the night. Valuntaite and co-authors focused on ozone measurements near two 330 kV high-voltage transmission lines arranged parallel to each other and found an excess of 2% in terms of atmospheric ozone near the lines [34]. Dirty or faulty insulators are known cases of arcing issues that may cause increased emissions of ozone. Ionized ozone may in turn lower air insulation properties, causing direct phase-to-phase or phase-to-metal cage discharge with considerable risks for infrastructural faults targeting distribution nodes. Detection of high levels of ozone can be considered an alarming condition, signaling impending faults. Figure 2 provides a graphical representation of the most relevant polluting issues concerning power lines. On the left, the major pollutants observed in the case of cables overheating or the corona effect are shown, while the case of cables burning is shown on the right.

### 2.2. Switchgears

While the association between ozone and electrical discharge is well known in the electrical engineering maintenance field, there is limited use of volatiles or particulate monitoring tools in electrical power transmission and distribution components. A notable example is reported in the 2020 study by Kakar [35]. There, an IoT system based on ozone sensors was developed for switchgear monitoring purposes. Anomalous conditions are screened and detected for predictive maintenance applications. This capability can be exploited to activate and optimize maintenance actions on switchgear components. On the other hand, for its direct impact on fugitive currents in insulators (also captured in IEC68150 [36] design recommendations), air pollution analysis and, in particular, aerosol concentration measurements were also used in [37] to assess status and predict faults in HV disconnectors, which was reviewed in [38].

### 2.3. Power Transformers

Power transformers are critical nodes of the electric power grid. These generally highly reliable systems are expected to have an useful operational lifetime of more than 25 years when operating temperatures are maintained between 65 °C and 95 °C, but this can be extended to up to 60 years when these conditions are preserved and proper maintenance regularly conducted. Gas generation in transformers is a relevant process connected to regular operation. Therefore, it is an important indicator of its health status [39].

In fact, for oil- and silicone-immersed transformers, slight damage to different parts of this infrastructure node can lead to an increase in dissolved gases that can be detected well in advance of a critical malfunction arising [40,41]. During normal operations, several analytes and compounds are released in the oil, including hydrogen (H_2_), methane (CH_4_), acetylene (C_2_H_2_), ethylene (C_2_H_4_), ethane (C_2_H_6_), carbon monoxide (CO), and carbon dioxide (CO_2_), most of which are found at low concentrations. Fast increases in their absolute or relative concentrations are anomalous events that can signal impending faults. Thermal failures, discharges, long-term exposure to electromagnetic fields, and the presence of water in the oil facilitate cracking and unpredicted chemical reactions, in turn generating H_2_, CO, and VOCs that dissolve into the oil itself and whose concentrations are related to the temperature, as shown in Table 1 [42]. Hence, continuous monitoring of the mixture of dissolved gas by DGA can be a powerful tool for predictive maintenance applications for these components [43]. Chemical sensors, including gas sensors, have also been reported for use in continuous monitoring applications for power transformers, and IEEE standards based on fuzzy logic or machine learning have been proposed to recognize the type of faults and their origin, such as excessive heat, arcing, or discharge phenomena [44]. Under specific conditions, peculiar compounds are released in oil, which are consequences of specific types of faults. Furfural, for example, is released because of insulation paper (winding) ageing. Therefore, furfural content or its proxies (primarily CO and CO_2_ and their ratio) can be exploited to obtain an estimate of the degree of polymerization in the winding insulation to predict the transformer’s lifetime, as attempted in [45], or transformer status, as attempted in [46,47]. Insulation deterioration can also be highlighted by carbon monoxide emissions. Acetylene may be a proxy signal for overheating, partial discharge, or even arcing (which in turn can also induce overheating).

**Figure 3:**
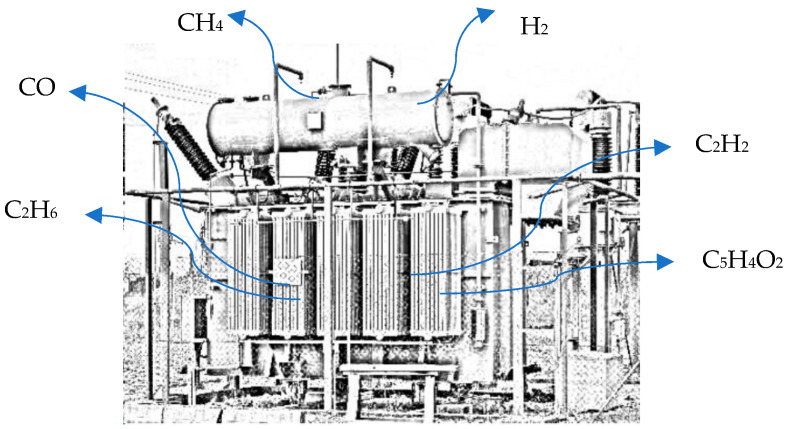
H_2_, Methane, CO, and the various VOCs that are detectable in the electric transformer environment.

Depending on the solubility of the target gas, fault detection can be best observed with gas sensors or by sampling of the oil reservoir, its headspace, or the transformer headspace itself. Hydrogen is, in fact, likely to easily escape the transformer and could be found at significant concentrations in the transformer headspace. This detection, however, will not provide information on the location of its production. Nitrogen, CO, and methane, which also share low solubility in the oil, are instead associated with the presence of both thermal and electrical faults [38], and their presence can be detected in the transformer headspace. Monitoring gas release in insulator fluids or headspace emissions is considered a critical issue for the preservation of these valuable assets. Regarding fugitive emissions, it has already been shown that distributed gas sensing may be an effective approach for their monitoring. In a study by Minglei et al., a complete solution following this approach was exploited and lab-tested for the monitoring of hydrogen emissions [48]. Figure 3 depicts the gas emissions that can be found in the transformer environment and whose relative concentrations can be correlated to specific faults.

### 2.4. Batteries

In modern power grids, batteries are a basic facility that provides inertia for system balance in the presence of distributed and variable energy sources. Unfortunately, they are prone to catastrophic malfunctions leading to fires and, more rarely, explosions [17]. In the automotive industry, new regulations now oblige that dangers to car passengers be signaled minutes before a dangerous event may occur [49]. Once started, fires can be particularly difficult to extinguish when the storage system size is significant because of the large DC power arc. The most common causes are electrical or mechanical runaways caused by overcharging, undercharging, or even short circuits caused by connected devices, including inverters. Harmful or dangerous gases are emitted before the fire takes place or in its initial stages during thermal runaways. Among them are HF at high volumes, which could be fatal when emitted in confined environments, carbon monoxide, methane, and VOCs. Ethylene carbonate, ethyl-methyl carbonate, diethyl carbonate, dimethyl carbonate, and propylene carbonate can be found in Li-ion batteries off-gassing from electrolytes and are considered the most relevant [50,51]. Actual ignition may render most of these components oxidized to harmless combustion by-products; however, since ignition must be avoided by any means, ignition denial may cause them to reach concentrations that easily lead to fatal outcomes for inhaling humans [52]. Even before thermal runaway may start, it has been demonstrated that CO2 emissions may signal this impending dangerous event, and chemical sensors may be used for alarming and for identifying the need for venting [53]. An increase in the concentration of hydrogen may also be considered as an early thermal runaway precursor due to unwanted electrolysis along with electrolyte vapor and gases produced by degassing of failing LIB batteries closer to thermal runaway events [54]. Because of the variety of emitted compounds and analytes, the use of a chemical multisensory device coupled with pattern recognition software for enhancing detection performance, rejecting false positives, and avoiding unwanted interference is recommended. In Table 2, the gaseous emissions so far reported by different authors and the main triggering events are summarized.

**Figure 4:**
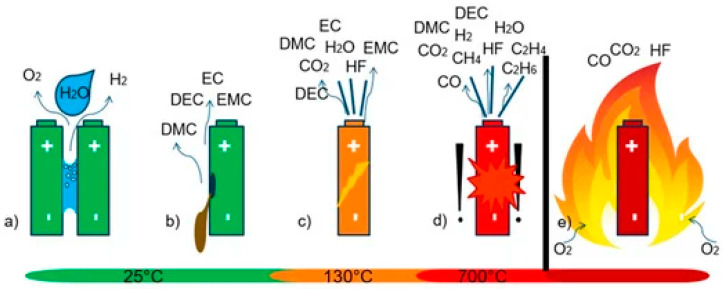
Gas emissions in failing batteries before unwanted electrolysis (**a**), in mechanical failure (**b**), and during thermal runaways (**c**,**d**) as conceptualized and depicted in [54]. The final ignition case is also considered (**e**). Refer to Table 2 for acronyms.

Finally, in Figure 4, a graphical representation of gas emissions in failing batteries is presented.

## 3. Commercial Systems

As mentioned above, during their operating lifetime, electric grid components may be sources of air polluting gases and aerosols. The effect is particularly severe up to the point of resulting in lethal concentrations in the case of malfunctions occurring in one or more equipment components. Continuous monitoring enables so-called data-based maintenance, a concept that assumes that component faults can be forecasted and that ageing processes can be monitored to properly schedule on-site intervention. Several companies are currently investing in research regarding methods for real-time monitoring of components of the electrical infrastructure to improve the reliability of power distribution systems. Honeywell (Charlotte, NC, USA) offers a large variety of gas sensors connected to the cloud that are directly remotely accessible for both reconfiguration and data transfer. The XNX, XCD, XRL, and S3000 series [60] all offer the possibility to monitor CO and CO_2_. The XNX series also allows for the monitoring of H_2_, while the XRL series can sample O_3_. The mentioned quantities are important data in the case of transmission and distribution lines in the proximity of which these gases can be found, and the presence of these gases could reveal an upcoming fault. General Monitors (Irvine, CA, USA) has in its portfolio the toxic gas detector TS4000 [61] with replaceable electrochemical cells, and the system is reconfigurable to satisfy any specific requirements. This system can be used for transmission and distribution line monitoring. Honeywell series 700-AS switches are equipped with two types of sensors: catalytic sensors and electrochemical sensors [62]. WoMaster has in-catalog sensors for H_2_, CO, CO_2_, O_3_, etc. They offer the possibility of putting together more sensors in a single case. Each sensor communicates with an RS485 bus connected to a gateway that exploits LORA communication or the Internet. Ad hoc WI-FI + LTE gateways and LORA NBIoT gateways to complete the network are also available [63]. The collected data can be sent to a proprietary server with no additional costs. These sensors are suitable for applications in the energy-transforming domain, as well as in transmission line applications or power station monitoring. Bosch proposes an all-in-one solution (BME688) that exploits artificial intelligence to obtain measurements of VOCs, VSCs, H_2_, CO, pressure, temperature, and humidity with a single sensor that can communicate the measurements to external systems [64]. This solution can be applied in the case of power transformers and battery monitoring. If a higher sampling frequency is required, Gas Sensing Solutions proposes the SprintIR^®^-R CO_2_ Sensor (CO_2_ Meter, Ormond Beach, FL, USA) for the measurement of carbon dioxide [65]. They claim that this sensor can take 50 measurements per second, ideal for high-speed monitoring of CO_2_ or locations where gas concentration might change rapidly. Honeywell proposes the Li-ion Tamer Gen3 sensor, which detects the electrolytic vapors of lithium-ion batteries. It also provides monitoring of the temperature and humidity of the environment [66]. Readings of gas, temperature, and humidity are sent via CanBus to the hub for storage or resending. These last sensors are useful for battery condition monitoring. Honeywell series 700 RL sensors are methane sensors that can be used in battery status monitoring. Methane is in fact emitted before a fire occurs during thermal runaway. In addition, CO_2_ sensors can be used for fire prevention purposes in the case of battery monitoring, and, in this case, the SprintIR^®^-R CO_2_ Sensor mentioned before can be used to quickly sample the air surrounding the battery to capture the rapid increase in the concentration of CO_2_. Schneider (Rueil-Malmaison, France) offers a closed solution for cable overheating, especially for power cabinets [67]. Vaisala (Vantaa, Finland) has been operating in the field of power measurement for many years. For power transformer condition monitoring there are several solutions, such as the OPT 100 [68], a DGA monitoring system claimed to require zero maintenance that is capable of auto-calibration.

## 4. Discussion

In this paper, a subset of research works highlighting the correlation between power grid electrical equipment operating conditions and specific gas emissions is reported and reviewed. It has been observed that in the case of transmission and distribution cables overheating, substantial concentrations of DOP and 2-EH are released into the air. Anomalous concentrations of ozone in the proximity of HV power lines, mainly due to the corona effect, have also been reported. However, only a limited subset of studies have analyzed such emissions in terms of their air pollution effect. Emitted ions are also known precursors of aerosol pollutants, but their concentration increase in the immediate surroundings is still disputed, although increased deposition due to HV has been clearly shown. To the best of our knowledge, the contribution of these emissions to air quality has not been considered in pollutant emissions inventories as they are expected to be, at least at present, too low to influence the balance. However, some authors have reported that the quantitative balance of ozone at the surface can be significantly affected near HVPLs. Ozone is present also in the case of the malfunction of switchgears and may itself contribute to further damages. Due to their extension, transmission and distribution power line cables should be carefully monitored in terms of their possible polluting emissions in case of cables burning when lethal limits for humans can be exceeded. During normal operation, oil-immersed power transformers release various gases both in the oil and eventually in air, including H_2_, CH_4_, C_2_H_2_, C_2_H_4_, CO, and CO_2_. The concentration of these gases has been observed to increase rapidly in the case of equipment anomalies. Electric batteries carry with them an intrinsic danger of overheating and thermal runaway that can lead to fire. In the time directly preceding the fire, VOCs, CO_2_, CH_4_, and H_2_ are released. Based on this evidence, some considerations can be made regarding the opportunity, perhaps the need, to enhance overall system reliability using condition monitoring, or in other words by electric system data-based maintenance operating by continuously monitoring the main asset of the system itself. Traditional approaches are based on redundancy, selectivity, and draw out technologies. Continuous condition monitoring allows system availability to be obtained at a reasonable cost. Installing IoT gas sensors along with power grid devices allows single components to communicate their status continuously. When a component reaches a predefined limit, it can be scheduled to be substituted at a time before the failure, thus avoiding environmental issues. In the case analyzed in this work, monitoring could be continuously performed on samples of the concentration of gases in proximity of the facility under observation. Several market solutions that allow for the design of an interconnected network of sensors are already available. Once all the useful information is collected, machine learning algorithms can be trained to recognize classified patterns of forthcoming failure. Furthermore, the grade of pollution can be estimated because, during normal operation, toxic gases are released.

## 5. Conclusions

The above-reviewed papers show that an operating electric transmission and distribution network may emit several atmospheric pollutants both in its normal everyday operation, such as in the case of HVPL cables because of the corona effect, and because of increasingly dangerous anomalies. Such emissions can, in general, be correlated to the specific fault a component is going to suffer. As a result, several IoT-based sensor systems that are suitable for implementing preventive fault detection are under investigation or even already on the market. It is worth noting that few papers have discussed the case of distribution networks, which is, on the contrary, quite relevant in terms of their possible effect on city environments. Moreover, quantitative investigations of the emitted gas concentrations are almost entirely absent. Future research should probably focus on these two aspects that are of relevance in terms of the expected massive electrification.

## Figures and Tables

**Table 1:** H_2_, CO, and VOCs observed during transformer operation, whose concentrations are related to the temperature and to the fault typology.

GAS	Fault Type	Concentration–Temp		
		<250 °C	250 °C < t < 350 °C	>350 °C
H_2_	Discharge, arcing	Low	Average (growing)	High
C_2_H_2_	Temp fault	Absent	Low	High
CO	Temp fault, cellulose	Absent	Low	High
C_2_H_2_	Arcing	Absent	Absent	High
CH_4_	Discharge	Low	High	Low
C_2_H_6_	Discharge	Absent	High	Low

**Table 2:** Anomalous electric battery gas emissions so far reported.

Expected Gas/Volatile Emission	Relevant Event	Reference
HF (Fluoridic Acid)	Thermal runaway, suppressed fire and explosion	[52,53,55]
H_2_ (Hydrogen)	Unwanted electrolysis, thermal runaway	[53,54,56,57,58]
CO (Carbon Monoxide)	Thermal runaway, fire and explosion	[52,53]
CO_2_ (Carbon Dioxide)	Thermal runaway, fire and explosion	[53,56,58]
EMC (Ethyl Methyl Carbonate)	Electrolyte loss or vaporization, thermal runaway, suppressed fire and explosion, unsuppressed fire and explosion (traces)	[50,51,52,54,55,56,58,59]
DMC (Diethyl Methyl Carbonate)	Electrolyte loss or vaporization, thermal runaway, suppressed fire and explosion, unsuppressed fire and explosion (traces)	[50,51,52,54,55,56,58,59]
EC (Ethylene Carbonate)	Electrolyte loss or vaporization, thermal runaway, unsuppressed fire and explosion (traces), suppressed fire and explosion	[50,51,52,54,55,56,58,59]
DEC (Diethyl Carbonate)	Electrolyte loss or vaporization, unsuppressed fire and explosion (traces), suppressed fire and explosion	[50,51,52,54,55,56,58,59]
C_2_H_4_ (Ethylene)	Thermal runaway	[54,58]
CH_4_ (Methane)	Thermal runaway	[54]
C_2_H_6_ (Ethane)	Thermal runaway	[54,56]
C_2_H_2_ (Acetylene)	Thermal runaway	[54]
C_4_H_10_ (Butane)	Thermal runaway	[54]
C_6_H_6_ (Benzene)	Unsuppressed fire and explosion (traces), suppressed fire and explosion	[52,53,54]
C_6_H_5_CH_3_ (Toluene)	Unsuppressed fire and explosion (traces), suppressed fire and explosion	[52]
(C₆H₅)₂ (Biphenyl)	Unsuppressed fire and explosion (traces), suppressed fire and explosion	[52]
C_3_H_4_O (Acrolein)	Unsuppressed fire and explosion (traces), suppressed fire and explosion	[52]

## Data Availability

Not applicable.
